# Living at the Extremes: Extremophiles and the Limits of Life in a Planetary Context

**DOI:** 10.3389/fmicb.2019.00780

**Published:** 2019-04-15

**Authors:** Nancy Merino, Heidi S. Aronson, Diana P. Bojanova, Jayme Feyhl-Buska, Michael L. Wong, Shu Zhang, Donato Giovannelli

**Affiliations:** ^1^Department of Earth Sciences, University of Southern California, Los Angeles, CA, United States; ^2^Earth-Life Science Institute, Tokyo Institute of Technology, Tokyo, Japan; ^3^Biosciences and Biotechnology Division, Physical and Life Sciences Directorate, Lawrence Livermore National Lab, Livermore, CA, United States; ^4^Department of Biology, University of Southern California, Los Angeles, CA, United States; ^5^Department of Astronomy – Astrobiology Program, University of Washington, Seattle, WA, United States; ^6^NASA Astrobiology Institute’s Virtual Planetary Laboratory, University of Washington, Seattle, WA, United States; ^7^Section of Infection and Immunity, Herman Ostrow School of Dentistry of USC, University of Southern California, Los Angeles, CA, United States; ^8^Department of Biology, University of Naples “Federico II”, Naples, Italy; ^9^Department of Marine and Coastal Science, Rutgers, The State University of New Jersey, New Brunswick, NJ, United States; ^10^Institute for Biological Resources and Marine Biotechnology, National Research Council of Italy, Ancona, Italy

**Keywords:** polyextremophiles, limits of life, astrobiology, habitability and astrobiology, extremophiles/extremophily, search for life

## Abstract

Prokaryotic life has dominated most of the evolutionary history of our planet, evolving to occupy virtually all available environmental niches. Extremophiles, especially those thriving under multiple extremes, represent a key area of research for multiple disciplines, spanning from the study of adaptations to harsh conditions, to the biogeochemical cycling of elements. Extremophile research also has implications for origin of life studies and the search for life on other planetary and celestial bodies. In this article, we will review the current state of knowledge for the biospace in which life operates on Earth and will discuss it in a planetary context, highlighting knowledge gaps and areas of opportunity.

## (Poly)Extremophiles Help Us Predict the Boundaries of Life

Over the past century, the boundary conditions under which life can thrive have been pushed in every possible direction, encompassing broader swaths of temperature, pH, pressure, radiation, salinity, energy, and nutrient limitation. Microorganisms do not only thrive under such a broad spectrum of parameters on Earth, but can also survive the harsh conditions of space, an environment with extreme radiation, vacuum pressure, extremely variable temperature, and microgravity (Horneck et al., 2010; Yamagishi et al., 2018). The definition of “extreme conditions” has strong anthropocentric criteria, rather than microbial criteria, and can be the cause of confusion (Rothschild and Mancinelli, 2001). When considering extremophilic (as opposed to extremotolerant) organisms, it is important to keep in mind that these are highly adapted organisms for the conditions considered and that the “extreme” condition constitutes the norm under which the organism is able to metabolically and biochemically operate. Moreover, there are myriad environments on our planet’s surface – and especially subsurface – that exhibit extremes in one or more physical or chemical condition. Therefore, extremophiles and, in particular, polyextremophiles (Capece et al., 2013) might be the most abundant lifeforms on our planet. In addition, if we consider that the current planetary surface conditions on Earth (such as mean temperature, redox state, and oxygenic atmosphere) have only occurred for a short period of time compared to the existence of life (Knoll, 2015), we might conclude that the extremophilic way of life has actually dominated the evolutionary history of life on our planet.

Over the past several decades, the isolation of culturable (poly)extremophiles and the identification of extreme microbial communities through various culture-independent approaches have provided key insights into the boundaries of life. Research on (poly)extremophiles has led to numerous advances in molecular biology and medicine (Babu et al., 2015; Coker, 2016; Durvasula and Rao, 2018), while simultaneously reshaping our understanding of the origins and evolution of life (Bertrand et al., 2015) and the potential for life on other planetary bodies (Schulze-Makuch, 2013). Several reviews have defined extremophiles (Table 1) (e.g., Rothschild and Mancinelli, 2001; Fang et al., 2010; Capece et al., 2013; Seckbach et al., 2013) and discussed the physiology and genetics of (poly)extremophiles in detail (e.g., chapters within *Polyextremophiles: Life Under Multiple Forms of Stress*, edited by Seckbach et al., 2013). To build upon these discussions, this paper will review the parameters that limit life, providing ranges under which life has been detected, herein defined as “boundary conditions.” We will then map the currently known boundary conditions of life on Earth to the possible conditions or theoretical space that life could occupy on Earth. Finally, we will explore the prospect of using this information for the search of life on other planetary bodies.

**Table 1:** Extremophiles nomenclature and ranges.

	Low → High^a^				
pH	Hyperacidophile	Acidophile	Neutrophile	Alkaliphile	Hyperalkaliphile
	(<pH 3)	(<pH 5)	(pH 5–9)	(>pH 9)	(>pH 11)
Temperature		Psychrophile	Mesophile	Thermophile	Hyperthermophile
		(<20°C)	(20–45°C)	(45–80°C)	(>80°C)
Salinity^b^		Non-halophile	Halotolerant	Halophile	Extreme halophile
		(<1.2%)	(1.2–2.9%; tolerate ≤ 14.6%)	(>8.8%)	(>14.6%, cannot grow < 8.8%)
Pressure			Piezotolerant or barotolerant	Piezophile or barophile	Hyperpiezophile or hyperbarophile
			(0.1–10 MPa)	(10–50 MPa)	(>50 MPa)
Water activity			Xerophile (a_w_ < 0.7)		
Polyextremophile	Tolerance or preference for multiple parameters combined				

*^*a*^The distinction between an extremotolerant microbe and an extremophile is based on the location of the optimum along the specific parameter range. See main text for discussion. ^*b*^Salinity expressed as percent of NaCl (w/v). Specific resistance to more chaotropic salts has been tested for some strains, for instance in the presence of MgCl_*2*_.*

## Parameters That Limit Life

Our knowledge of life is based on the observable and measurable phenomena that occur on Earth, and is therefore limited to this instance of life. However, the laws of chemistry and physics have universal principles which enable us to extrapolate to the conditions under which life could survive elsewhere. These principles suggest that life requires a liquid solvent, an energy source, and building blocks (Schwieterman et al., 2018).

While the bulk abundance of (inorganic) building blocks appears not to be a factor limiting the distribution of life on Earth (with subsurface environments as a possible exception, e.g., Hoehler and Jørgensen, 2013) and, potentially, other planetary bodies, the availability of a solvent is considered to be a key factor. While the potential for other liquid solvents to sustain extraterrestrial life is discussed in detail elsewhere (Schwieterman et al., 2018 and references therein), water is considered the most likely liquid solvent because of its cosmic abundance and physicochemical properties (Michiels et al., 2008; Schwieterman et al., 2018). Water, especially the availability of liquid water, appears to be the main factor controlling the dimensions of the biospace for life on Earth (i.e., the parameter space occupied by life). Liquid water acts both as a solvent and a reactant/product in biochemical reactions, and its numerous unique physicochemical properties have profoundly shaped the emergence and evolution of life on our planet. As discussed in this review below, water activity appears to be the single key parameter controlling the biospace of Earth’s life, and numerous other parameters limiting life (e.g., temperature and salinity) are, in fact, acting on the availability of water. At the ecosystem level, water can indirectly influence the variation of key physicochemical conditions, which in turn controls microbial community composition and diversity, profoundly influencing geobiochemical cycling (*sensu* Shock and Boyd, 2015).

Life also needs a source of energy to power chemical reactions, and redox chemistry appears to be universal (Jelen et al., 2016). Physicochemical gradients create non-equilibrium redox conditions that have played an important role in the origins, evolution, and diversity of life. Redox and proton gradients were likely the two main mechanisms involved in the origins of life, initiating the necessary energy flux to drive metabolism and growth (Lane et al., 2010; Lane and Martin, 2012). Therefore, the current search for life’s limits has extended beyond temperature, pH, pressure, salinity, and radiation gradients (each parameter discussed in their respective sections) and also includes the possible energetic and nutrient limits of life (discussed in Hoehler and Jørgensen, 2013; LaRowe and Amend, 2015; Jones et al., 2018).

The parameters discussed herein (temperature, pH, pressure, salinity, and radiation) correlate with each other and can influence the availability of nutrients and energy sources. Depending on the environment, certain parameters can more strongly influence microbial diversity over others, such as temperature in geothermal waters (Sharp et al., 2014), pH in soil communities (Rousk et al., 2010), salinity in saline lakes (Yang et al., 2016), and water content in dry climates (Dose et al., 2001). On the nano- and micro-scale level, the two most important factors are likely water activity and pH, which influence the chemiosmotic, energy-generating gradient at the cell level (Lane et al., 2010; Lane and Martin, 2012). In contrast, parameters that influence the macro-scale level vary with the ecosystem. For example, temperature plays a significant role in geothermal environments and influences such processes as water-rock interactions and degassing (Nordstrom et al., 2005; Fouke, 2011; Cole et al., 2013; Price and Giovannelli, 2017). Water-rock interactions can then impact microorganisms by limiting the availability of trace elements and electron donors/acceptors.

**FIGURE 1:**
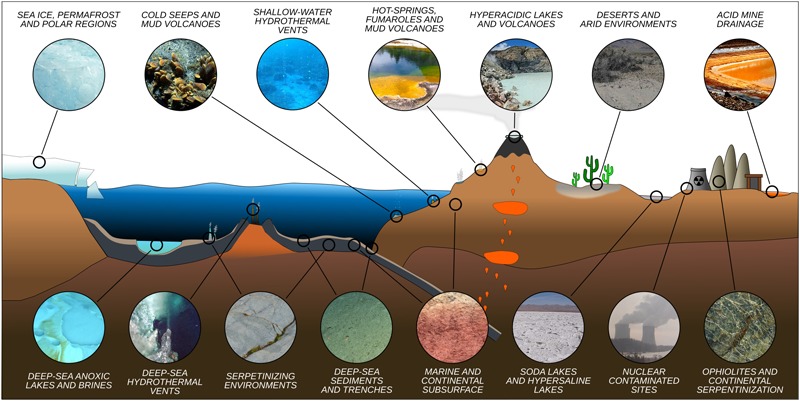
Representative idealized cross section of Earth’s crust showing the diversity of extreme environments and their approximate location.

**FIGURE 2:**
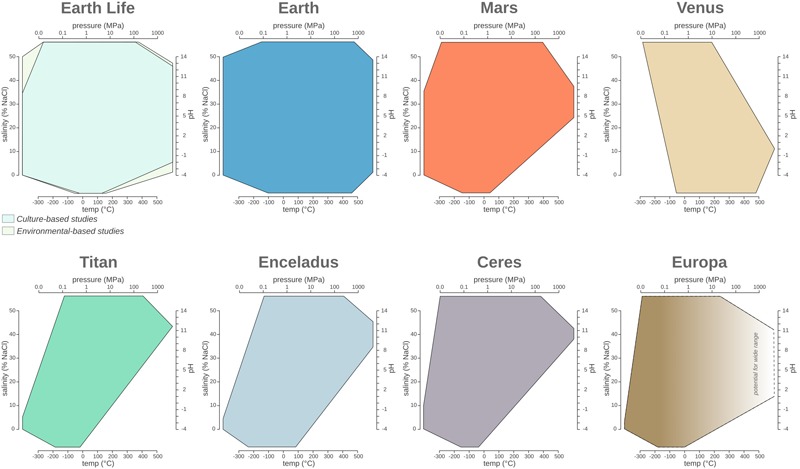
The temperature, pressure, pH, and salinity boundaries observed for life on Earth compared to the phase space observed on planetary bodies discussed in the main text. Polygon charts are designed to represent ranges in multidimensional space. Each edge represents the range for the specific variables Single values (e.g., when min = max) are represented by a single vertex on an axis, while missing values (e.g., NA or NR) are represented by the absence of the corresponding polygon edge on the corresponding axis.

Microorganisms have been detected in a variety of extreme environments (Figure 1), virtually in any location where liquid water is available for life to use. This demonstrates that life can adapt to a wide range of parameters (Figure 2). It is therefore imperative to determine the minima and maxima for each parameter, and even more importantly, to understand their combined effects, in order to evaluate the limits of Earth’s life and advance our understanding of the potential for life elsewhere.

### Acidity and Alkalinity

Extremely low and high pH environments have been observed for different ecosystems on Earth (Table 2). Extreme pH values were observed for ecosystems contaminated by mining waste, with current extremes reported from Iron Mountain (Shasta County, CA, United States) (pH -3.6) (Nordstrom et al., 2000) and Gorka Lake (Chrzanow region, Poland) (pH 13.3; Czop et al., 2011). While there has yet to be any microbial community studies or isolation attempts for Gorka Lake, to the best of our knowledge, microbial communities have been explored at Iron Mountain (Baker and Banfield, 2003), with several microorganisms isolated [e.g., *Thermoplasmales* (Edwards et al., 2000), *Acidithiobacillus ferrooxidans* (Schrenk et al., 1998; Kelly and Wood, 2000), and *Leptospirillum ferrooxidans* (Schrenk et al., 1998)]. Despite this, there are currently no cultured or isolated microorganisms which can be grown at either of the listed extremes. Currently, the most extreme acidophile and alkaliphile can survive at pH 0 and pH 12.5, respectively (pH_opt_ 0.7 and 11) (Table 3). The lowest pH_min_ -0.06 was observed for two hyperacidophilic Archaea known as *Picrophilus oshimae* and *P. torridus* (pH_opt_ 0.7), isolated from a solfataric hot spring in Noboribetsu (Hokkaido, Japan) (Schleper et al., 1996). These heterotrophic and aerobic polyextremophiles can also withstand temperatures of up to 65°C (*T*_opt_ = 60°C, *T*_min_ = 47°C), potentially through increased cyclization of their tetraether membrane lipids as a generalized response to pH, temperature, and nutrient stress (Feyhl-Buska et al., 2016). Other thermoacidophiles also include those species within the genus *Sulfolobus*, in which several isolates are known to be genetically tractable (Quehenberger et al., 2017). In comparison to extreme acidophily, the highest pH_max_ of 12.5 was observed for an alkaliphilic, aerobic, mesophilic bacterium known as *Serpentinomonas* sp. B1 (pH_opt_ 11), isolated from a terrestrial serpentinizing system, The Cedars (CA, United States) (Suzuki et al., 2014). Although there is a report of the highest pH_max_ 13 held by *Plectonema nostocorum* (Kingsbury, 1954), this has not been further confirmed. The largest pH range, as compared to other isolated microorganisms, was observed for *Halomonas campisalis* (pH_range_ 6–12), a haloalkaliphilic bacterium isolated from a soda lake (Soap Lake, WA, United States) (Mormile et al., 1999; Aston and Peyton, 2007) (Table 4).

**Table 2:** Environmental boundary conditions for different Earth ecosystems.

Biome	Temperature	pH	Pressure	Salinity	References
	(°C)		(MPa)	(% NaCl)	
Soda lakes	-0.5–83	6.9–11.2^a^	nr	0.64–37.1	Jones et al., 1977; Tambekar et al., 2010; Kavak and Karadogan, 2012; Pontefract et al., 2017
Terrestrial hot springs/geothermal waters	15–270	0.02–9.8	0.1–7.2^b^	0.0002–saturation	Delmelle and Bernard, 1994; Namsaraev et al., 2003; Taran, 2009; Chan et al., 2017; Qi et al., 2017
Polar environments	-98.6–24.3	4.6–9.6	0.1–35.5^c^	0–40.2	Siegert et al., 2001; Aislabie et al., 2006; Samarkin et al., 2010; Dickson et al., 2013; Scambos et al., 2018
Deep-sea floor and trenches	-1.9–13.8^d^	7.3–8.1	2.1–112	3.4–3.9	Mantyla and Reid, 1983; Emeis et al., 1996; Danovaro et al., 2010
Deep-sea hydrothermal vents	<1^e^–464	4–11	2.1–50.7	0.1–8	Koschinsky et al., 2008; Konn et al., 2009; McDermott et al., 2018
Deep hypersaline anoxic basins	10–65	5.4–8.6	2.1–40.5	4–50^f^	Karbe, 1986; Yakimov et al., 2013, 2015; Mapelli et al., 2017; Merlino et al., 2018
Subsurface ecosystems	3.25–<400^g^	∼1–12.8	<800^h^	0.05–saturation^i^	Becker et al., 1984; Roadcap et al., 2006; Waldron et al., 2007; Pankova and Konyushkova, 2013; Lechmann et al., 2014; Frank et al., 2016; Prieto et al., 2017
Deserts^j^	-19.4–70	6.8–10	nr	0.02–30.8	El-Demerdash et al., 1995; James et al., 2005; Dion et al., 2008; Pandit et al., 2015; Holm et al., 2017
Serpentinite-hosted systems^k^	10–365	2.8–12.6	<900^l^	0.03–49.68^m^	Scambelluri et al., 1997; Van Dover et al., 2001; Mottl et al., 2003, 2004; Schrenk et al., 2013; Suzuki et al., 2013
Mine drainage	1^n^–47	-3.6–13.3	6–14	0.008–7.6	Nordstrom et al., 2000; Tutu et al., 2008; Czop et al., 2011; Miettinen et al., 2015

*nr, not reported. ^*a*^Highest pH from brine in a causeway on Lake Magadi (Jones et al., 1977). ^*b*^Pressure of geothermal spring along Moxi fault (Western Sichuan plateau, China) (Qi et al., 2017). ^*c*^The highest reported pressure is for Lake Vostok, Antarctica, covered by 4 km of ice (Siegert et al., 2001). ^*d*^Highest deep-sea temperature is from the Mediterranean Sea (Danovaro et al., 2010). Geothermal influenced deep-sea sediments are not considered here. ^*e*^pH of venting fluids from the deep-sea arc volcano NW Rota-1, Mariana Arc (Resing et al., 2007). ^*f*^Includes both thalassic (NaCl dominated) and athalassic (MgCl_*2*_ dominated) anoxic basins. ^*g*^Low temperature of 3.25°C is an uncertain lower bound of *in situ* temperatures at Hole 395B (North Pond, Mid-Atlantic Ridge) (Becker et al., 1984); High temperature of 400°C is theoretical upper temperature of the crust-mantle boundary. ^*h*^Estimated highest pressure at the India Asia collisional system (Lechmann et al., 2014). ^*i*^Saturation observed in halite deposits (Jaakkola et al., 2016). ^*j*^Does not include polar region deserts. ^*k*^Includes both marine and terrestrial serpentinization sites. ^*l*^Subduction zone at Conical Seamount (Mariana Forearc) (Mottl et al., 2004). ^*m*^Highest salinity occurred in salt inclusions of Erro-Tobbio, Italian Western Alps (Scambelluri et al., 1997). ^*n*^Low temperature reported for the Gorka Pit Lake, Poland (Czop et al., 2011). ^*o*^Hydrostatic pressure of fluid fracture network in Pyhäsalmi mine, Finland (Miettinen et al., 2015).*

**Table 3:** Limits of life as identified by (poly)extremophilic organisms in pure cultures.

Strain	Domain	Extremophile	Isolation	Temperature	pH	Pressure	Salinity	Water	References
		Type	ecosystem	(°C)		(Mpa)	(%)	activity (a_w_)	
*Picrophilus oshimae* KAW 2/2	*Archaea*	Hypercidophile	Hot springs, Solfataras	47–65 (60)^a^	**-0.06–**1.8 (0.7)	nr	0–20	nr	Schleper et al., 1995, 1996
*Serpentinomonas* sp. B1	*Bacteria*	Alkaliphile	Serpentinizing system (water)	18–37 (30)	9–**12.5** (11)	nr	0–0.5 (0)	nr	Suzuki et al., 2014
*Methanopyrus kandleri* 116	*Archaea*	Hyperthermophile	Deep-sea hydrothermal vent	90–**122** (105)	(6.3–6.6)	0.4–40	0.5–4.5 (3.0)	nr	Takai et al., 2008
*Planococcus halocryophilus* Or1	*Bacteria*	Halopsychrophile	Sea ice core	**-15**–37 (25)	6–11 (7–8)	nr	0–19 (2)	nr	Mykytczuk et al., 2012, 2013
*Halarsenatibacter silvermanii* SLAS-1	*Bacteria*	Haloalkaliphile	Soda lake	28–55 (44)	8.7–9.8 (9.4)	nr	20–35 (**35**)	nr	Oremland et al., 2005
*Thermococcus piezophilus* CDGS	*Archaea*	Piezothermophile	Deep-sea hydrothermal vent	60–95 (75)	5.5–9 (6)	0.1–**125** (50)	2–6 (3)	nr	Dalmasso et al., 2016
Haloarchaeal strains GN-2 and GN-5	*Archaea*	Xerophile	Solar salterns (brine)	nr	nr	nr	nr	**0.635**	Javor, 1984

*^*a*^Data presented as range (optimum) for each parameter. nr, not reported in the original publication. Current limits are highlighted in bold.*

**Table 4:** Examples of notable (Poly)extremophiles and their physiological requirements.

Strain	Domain	Extremophile	Isolation	Temperature	pH	Pressure	Salinity	References
		type	ecosystem	(°C)		(MPa)	(%)	
*Acidianus infernus* So4a	Archaea	Acidothermophile	Solfatara crater	65–96 (90)	1–5.5 (2)	na	0.2 (na)	Segerer et al., 1986
*Colwellia piezophila* ATCC BAA-637	Bacteria	Piezopsychrophile	Deep-sea	4–15 (10)	7 (na)	40–80 (60)	na (3)	Nogi et al., 2004
*Halomonas campisalis* MCM B-365	Bacteria	Hyperalkaliphile	Soda lake	4–50 (30)	6–12 (9.5)	na	1.1–26.3 (8.9)	Aston and Peyton, 2007
*Oceanobacillus iheyensis* HTE831	Bacteria	Alkaliphile, piezotolerant, and halotolerant	Deep-sea (mud)	15–42 (30)	6.5–10 (7–9.5)	0.1–30	0–21 (3)	Lu et al., 2001
*Anoxybacillus pushchinensis* K1	Bacteria	Alkalithermophile	Manure	37–66 (62)	8–10.5 (9.5)	na	<3 (na)	Pikuta et al., 2000
*Actinopolyspora righensis* H23	Bacteria	Halophile	Saline soil	20–40 (28–32)	5–8 (6–7)	na	10–30 (15–25)	Meklat et al., 2013
*“Geothermobacterium ferrireducens”* FW-1a	Bacteria	Hyperthermophile	Obsidian Pool, Yellowstone National Park	65–100 (85)	na	na	0 (na)	Kashefi et al., 2002
*Shewanella piezotolerans* WP3	Bacteria	Piezophile	Deep-sea	0–28 (15–20)	6–8 (7)	0.1–50 (20)	1–7.2 (3–4)	Xiao et al., 2007
*Colwellia* sp. MT-41	Bacteria	Piezopsychrophile	Deep-sea	2 (na)	6.8 (na)	51.8–103.5 (69)	na	Yayanos et al., 1981
*Pedobacter arcticus* A12	Bacteria	Psychrophile	Tundra (soil)	4–25 (18)	6–9 (7)	na	0–2 (0)	Zhou et al., 2012
*Thermococcus gammatolerans* EJ3	Archaea	Thermophile and radiation-tolerant	Hydrothermal vent (chimney)	55–95 (88)	na (5.5–6.5)	na	(20)	Jolivet et al., 2003
*Deinococcus radiodurans* R1	Bacteria	Vacuum- and radiation-tolerant	Spoiled canned meat	Mars-like conditions, vacuum, UV and space radiation				De Vera et al., 2012
*Cryomyces antarcticus* MA5682	Fungi	Vacuum- and radiation-tolerant	Antarctica	Mars-like conditions, vacuum, UV and space radiation				De Vera et al., 2012
*Deinococcus geothermalis* DSM 11300	Bacteria	Xerotolerant	Hot spring	30–55 (47)	5–8 (6.5)	na	na	Frösler et al., 2017
*Halobacterium salinarum* NRC-1	Archaea	Xerotolerant, vacuum- and radiation-tolerant	Bore core from a salt mine	42 (na)	na	na	25	Kottemann et al., 2005

The pH has a significant effect on microorganisms and microbial consortia, ranging from the nano- to macro-scale level. All microorganisms must maintain a near neutral cytoplasmic pH to enable cellular functions for survival and metabolism (Krulwich et al., 2011; Jin and Kirk, 2018). The cytoplasmic pH of acidophilic bacteria is ∼6.0 while alkaliphilic bacteria have a cytoplasmic pH around 7.2–8.7 (Krulwich et al., 2011). For more information on the molecular mechanisms behind pH homeostasis, Krulwich et al. (2011) provide a detailed review. The homeostasis of protons (and other ions) through various transporters, including the ion-utilizing ATP synthase, was likely one of the first functions to develop within the earliest cells (Lane and Martin, 2012). Indeed, chemiosmosis is a property of both archaeal and bacterial cells (Lane et al., 2010). In addition to intracellular pH, microorganisms can excrete organic metabolites, such as lactic acid or acetic acid, thereby changing the immediate, surrounding pH (Zhang et al., 2016). Many acidophiles also have organic acid degradation pathways to prevent proton uncoupling by organic acids (Baker-Austin and Dopson, 2007). It has been demonstrated both in natural settings and laboratory cultures that microorganisms can significantly alter their environmental pH as a result of metabolic reactions. For example, sulfide, thiosulfate, and elemental sulfur oxidizers secrete sulfate and protons as by-products, significantly acidifying their environment. This ability is used industrially for the bio-leaching of sulfide ore deposit (Olson et al., 2003; Rohwerder et al., 2003) and it is largely responsible for the low pH of acid mine drainage fluids and other acidic environments. Recent work by Colman et al. (2018) suggests that thermoacidophilic archaea and the acidity of their habitats co-evolved after the evolution of oxygenic photosynthesis (since oxygen is used as primary electron acceptor in the metabolisms), showing a significant example of niche engineering and geosphere-biosphere coevolution. All together, these findings suggest that pH can be metabolically controlled either at the intracellular or local level, as compared to temperature, radiation, salinity, and pressure.

On the macro-scale level, pH can dominate as the main parameter affecting microbial community composition and abundances. Several studies demonstrate that pH affects microbial community diversity more than any other parameter tested (e.g., Lauber et al., 2009; Rousk et al., 2010; Xiong et al., 2012; Kuang et al., 2013; Zhalnina et al., 2014). For example, distinct microbial communities were observed with changes in pH (pH_range_ 1.9–4.1), in which the genus *Ferrovum* dominated at higher pH while the phyla *Alphaproteobacteria, Gammaproteobacteria, Nitrospirae*, and *Euryarchaeota* were present at lower pHs (Kuang et al., 2013). Similarly, bacterial community composition changed with increasing pH in alkaline sediments of a Tibetan plateau (pH_range_ 6.88–10.37) (Xiong et al., 2012). Changes in community composition are likely derived from the range in which microorganisms can survive (Fernández-Calviño and Bååth, 2010). Most cultured microbes live within a narrow pH range of three to four units (Rosso et al., 1995), although some exceptions occur [e.g., fungal isolates can grow over five to nine pH units (Wheeler et al., 1991; Nevarez et al., 2009)]. Moreover, it has been suggested that archaeal (Kuang et al., 2013) and fungal communities (Rousk et al., 2010) may be less affected by changes in pH compared to bacteria.

### Salinity and Water Activity

Salinity has a significant impact on microbial community composition (Lozupone and Knight, 2007; Swan et al., 2010). Saline environments comprise a large portion of the Earth and range from the marine environment (∼3–4% salinity), hot springs (up to 10.5% salinity), and to soda lakes (up to 37.1% salinity), and even salt inclusions [up to 49.7% salinity (Scambelluri et al., 1997)] (Figure 2 and Table 2). Salinity can also vary significantly on smaller scales, for example, in tidal pools (Morris and Taylor, 1983), or on salt mineral grains due to water deliquescence (Davila et al., 2008). A wide range of different ions, including Na^++^, Cl^-^, SO_4_^2-^, Ca^2+^, and Mg^2+^ (Oren, 2013) can contribute to total salinity in the environment. The ionic composition can significantly influence water activities, especially in the presence of high concentrations chaotropic salts, like in the athalassic deep-sea hypersaline anoxic basins of the Mediterranean Sea (Yakimov et al., 2015). In addition, water availability in terrestrial saline environments is further influenced by precipitation rates relative to evaporation, resulting in increasing concentration of salts (Finlayson et al., 2018).

The salinity range and optimum for cultivable and isolated microorganisms is between 0 and 35%. The current highest salinity record holder is *Halarsenatibacter silvermanii* strain SLAS-1^T^, isolated from the alkaline hypersaline Searles Lake (CA, United States) (salinity_opt_ 35% NaCl) (Blum et al., 2009). Halophiles are found in all three domains of life (DasSarma and DasSarma, 2017). Current hyperhalophiles in culture include bacteria and archaea which can grow over a salinity of ∼15% NaCl. There are also polyextremophiles, for example, the bacterium *Halomonas campisalis* (Table 4), isolated from a soda lake (Soap Lake, WA, United States) is a moderate halophile and alkaliphile (salinity_opt_ = 8.8%, pH_opt_ = 9.5) and can tolerate extreme pH up to 12 and salinities up to 26.3% (Mormile et al., 1999; Aston and Peyton, 2007).

Halophiles achieve the necessary osmotic balance by one of two strategies: (1) accumulating K^+^ in the cytoplasm as a ‘salt-in’ strategy or (2) excluding salts by synthesizing compatible organic solutes, such as polyols, amino acids, sugars, and betaines. The ‘salt-in’ strategy has been identified only in a few halophiles (e.g., *Salinibacter* and *Halanaerobiales*) which require KCl to have functional proteins. In contrast, many microorganisms that utilize the salt exclusion strategy can tolerate a wider range of salt concentrations due to the production of organic solutes to counter the concentration of salts (Oren, 2011). The necessary energy needed to maintain osmosis, and the thermodynamics of surviving under saline conditions have been thoroughly discussed by Oren (2011).

Many microorganisms in saline environments must also adapt to low water activity (the mole fraction of water) and increased radiation (discussed in section “Radiation”). Although salts can lower the freezing point of water, saturated salt solutions have low water activity. Water activity is the only other parameter, aside from pH and salinity, that some microorganisms can regulate through the production of metabolites capable of storing or attracting water (e.g., proteins and polysaccharides from EPS) (Frösler et al., 2017). The theoretical water activity minima for halophilic archaea and bacteria is 0.611 a_w_ while it is 0.632 a_w_ for fungi (Stevenson et al., 2015). In comparison, the water activity of NaCl saturated solutions is estimated to be 0.755 a_w_ while pure water is 1 a_w_ (Hallsworth et al., 2007; Stevenson et al., 2015).

The theoretical water activity limit has been surpassed by microbial life. When there are high concentrations of the chaotropic MgCl_2_ or CaCl_2_, the water activity is lowered even more (e.g., 0.3 a_w_ for a saturated MgCl_2_ solution). For example, environmental surveys reported microbial communities in the brines of two athalassic deep-sea hypersaline anoxic basin (DHAB), Discovery (MgCl_2_ ≥ 5 M, T = 14.5°C) (Van Der Wielen et al., 2005) and Kryos Basin (saturated MgCl_2_, ∼0.4 a_w_, *T* = 16.5°C) (Alcaide et al., 2015; Steinle et al., 2018), both located in the Mediterranean Sea. The Kryos Basin microbial community, located in the brine, consisted of active sulfate-reducers, with sulfate reduction reaching up to 460 μmol/kg-day (Steinle et al., 2018). In contrast to the DHABs, microbial life has yet to be shown to exist in a CaCl_2_-dominated brine with up to 474 g/L total dissolved salts (Don Juan Pond, Antarctica) (Oren, 2013). This is likely due to both extreme temperature and salinity conditions, as Don Juan Pond is an unfrozen lake (pH 4.6) with an average depth of 11 cm and temperatures reaching below -36°C (*T*_max_ ∼ 20°C) (Torii et al., 1981; Samarkin et al., 2010; Dickson et al., 2013). The estimated water activity in Don Juan Pond is likely below 0.45 a_w_ (Oren, 2013) but could be between 0.28 a_w_ (25°C) to 0.61 a_w_ (–50°C), as estimated for a CaCl_2_-dominated brine with antarcticite (CaCl_2_⋅6H_2_O) precipitation (Toner et al., 2017).

### Temperature

The temperature on Earth’s surface ranges from -98.6 to 495°C [ultra-cold locations in East Antarctica (Scambos et al., 2018) and extremely hot deep-sea hydrothermal vents (McDermott et al., 2018)], with much higher temperatures possible in magma influenced subsurface environments (Table 2). Fluid temperatures above 100°C are possible whenever the combination of hydrothermal or magmatic activity is present together with high pressure, for example, in the deep subsurface near volcanoes or at deep-sea hydrothermal vents. In the absence of geothermal influence, the highest surface temperature reported on Earth is ∼71°C, in the Lut Desert (Iran) (Mildrexler et al., 2011). The current temperature extreme that microbial life can survive extends from -25°C (*T*_min_, *Deinococcus geothermalis* DSM 11300) (Frösler et al., 2017) to 130°C (*T*_max_, “*Geogemma barossii”* 121) (Kashefi and Lovley, 2003) (Table 4). Around -26°C to -10°C, microbial cells will likely become vitrified (without intracellular freezing), enabling cells to survive low temperatures (Clarke et al., 2013). The temperature range in which microorganisms are reported to be metabolically active is currently between -20°C (an enrichment culture from the Siberian permafrost soil) (Rivkina et al., 2000) and 122°C (*Methanopyrus kandleri* 116; Takai et al., 2008). In comparison, the lowest temperature in which a pure culture isolate is capable of growing is -15°C with 18% salinity (*Planococcus halocryophilus* Or1; Mykytczuk et al., 2012, 2013).

The upper temperature of life has been raised several times in the past 50 years of research (Brock and Freeze, 1969; Ferrera and Reysenbach, 2007), and current environmental and theoretical studies suggest that the upper limit of life might lay near ∼150°C, due primarily to the instability of macromolecules above this temperature. Similarly, thermodynamic considerations suggest that life might be impossible below -40°C (Price and Sowers, 2004), thus the current theoretical boundaries for life are -40°C to 150°C. It is still possible, however, that the boundary conditions of life might extend past these limits, and the surpassing of previous historical theoretical limits suggest that future studies might unveil unexpected adaptation strategies.

Extreme temperature adaptations by psychrophiles and thermophiles generally involve either high saline or pressure conditions. High saline, cold environments enable the growth of halopsychrophiles (Deming, 2007). Liquid inclusions in sea ice are due to the high concentrations of salts, which lower the freezing point of water, and this liquid fraction can still be observed at -40°C (theoretical seawater eutectic temperature is -55°C) (Deming, 2007). Microbial consortia are likely to inhabit subzero brine veins, especially those surrounding soil particles, where salts and organic materials (e.g., the microbially produced extracellular polymeric substances or EPS) are concentrated. Indeed, the majority of active bacteria and archaea observed in Arctic wintertime sea-ice cores at -20°C were all particle-associated (Junge et al., 2004). In contrast to halopsychrophiles, there are very few halothermophiles, with a combined temperature range of 17–70°C (*T*_opt_ = 50–65°C) and salinity range 2.9–29.2% (salinity_opt_ = 11.7–26.3% NaCl) (Mesbah and Wiegel, 2005). Several hyperthermophiles (growth at >80°C) must grow at high pressure conditions because high pressure allows water to remain liquid at higher temperatures, with an upper theoretical limit of 407°C at 29.8 MPa pressure (Koschinsky et al., 2008; McDermott et al., 2018). Hyperthermopiezophilic microorganisms, such as *Methanopyrus kandleri* strain 116 (Takai et al., 2008) and “*Geogemma barossii”* strain 121 (Kashefi and Lovley, 2003) (Table 4), are able to maintain cell structural integrity due to the contrasting effects of high temperature and high pressure.

Macro-scale temperature gradients demonstrate the influence of temperature on microbial community composition within an ecosystem (Purcell et al., 2007; Miller et al., 2009; Everroad et al., 2012; Cole et al., 2013; Sharp et al., 2014). In this regard, the effect of increasing temperature gradients, especially in geothermal-influenced environments, have been studied to greater extent compared to decreasing temperature gradients. In general, the community complexity decreases with increasing temperatures on the scale of centimeters to meters. For example, the soil microbial community of Tengchong Geothermal Field (China) shifted toward lower diversity with increasing temperatures (50–90.2°C and 32–36 MPa) and became dominated by Archaea (Li et al., 2015). Similar patterns have been also reported for deep-sea and shallow-water hydrothermal vents (Flores et al., 2012; Giovannelli et al., 2013). Temperature gradients likely have more influence on the microbial community of geothermal environments (Sharp et al., 2014), as compared to other environments (e.g., soil), where pH and salinity have been shown to be the dominant factor (see sections “Acidity and Alkalinity” and “Salinity and Water Activity”).

### Pressure

As mentioned above, pressure influences microbial growth, especially under extreme temperatures. On Earth’s surface, pressure ranges from 0.1 to 112 MPa (Table 2), with higher pressures observed at subduction zones (e.g., 900 MPa at the top of a subducting plate, Mariana Forearc; Mottl et al., 2004) and subsurface environments (e.g., Miettinen et al., 2015). It is estimated that microbial life could be supported at subduction zone forearcs with pressures ∼340 MPa (Plümper et al., 2017). Several piezophiles and piezotolerant microorganisms have been isolated from deep-sea locations (Table 3), and the current record holder is *Thermococcus piezophilus*, a thermophilic Archaeon able to survive up to 125 MPa (*P*_opt_ = 50 MPa, *P*_growth range_ = 0.1–125 MPa) (Dalmasso et al., 2016). Piezophiles have lower generation times at higher pressure than at atmospheric pressure (Bartlett et al., 2007), and considering the average depth of the ocean is 3,800 m (average pressure 38 MPa), with bottom temperatures between 0 and 3°C, there is likely a vast number of uncultured piezophiles across a range of temperatures, including a vast majority of psychropiezophiles (Alazard et al., 2003; Fang et al., 2010). Despite the small number of strict piezophiles currently in culture, environmental studies suggest that life can easily accommodate high pressures, and studies on piezotolerant strains have demonstrated that life can survive brief exposures up to 2,000 MPa (Sharma et al., 2002; Vanlint et al., 2011). Under these extreme conditions, cells have been shown to be metabolically active in fluid inclusions found in ice-VI crystals within diamond anvil cells (Sharma et al., 2002).

(Hyper)piezophiles have adapted to extreme pressures through various strategies. In particular, the cell membrane is packed with more unsaturated fatty acids to increase membrane fluidity at high pressures. Other adaptations could include upregulation chaperone-encoding genes, modification of the respiratory chain, expression of different porins, and production of osmolytes (Oger and Jebbar, 2010; Jebbar et al., 2015). Several detailed reviews on piezophile adaptation strategies are available, including Fang et al. (2010), Oger and Jebbar (2010), Picard and Daniel (2013), and Jebbar et al. (2015).

In contrast to high pressure environments, the low pressure found at high altitude in mountain formations (0.0033 MPa at the summit of Mount Everest) is unlikely to affect microbial survival *per se*, and the lowest pressure is found in space vacuum or low Earth orbit (10^-13^ to 10^-10^ MPa) (Horneck et al., 2010). Despite this, several prokaryotes, fungi, and lichen can survive exposure for several months to years under space conditions (Horneck et al., 2010; De Vera et al., 2012; Onofri et al., 2018; Yamagishi et al., 2018), due to sporulation or formation of biofilms (Frösler et al., 2017). It is possible that the top layer of a biofilm protects the lower layers, enabling the survival of microorganisms under space conditions. For example, *Deinococcus aetherius ST* survived a 1-year exposure to space conditions only when ≥500 μm cell layer was utilized (Yamagishi et al., 2018). However, longer exposure to space vacuum can cause detrimental effects, such as dehydration and DNA denaturation, and likely requires pre-dried microbial spores or biofilm within a protective substance (e.g., sugars or buffer salts). For more information, Horneck et al. (2010) have written a detailed review on space condition effects on microorganisms.

The effects of pressure on microbial community composition can be observed most obviously in deep-sea environments. However, it is likely that other parameters dominate as the major contributors to community composition and abundances, such as salinity, temperature, oxygen concentrations, and UV radiation (Amend and Shock, 2001; Phoenix et al., 2006; Walsh et al., 2016), rather than pressure. In contrast to deep-sea environments, there have been few studies examining the microbial community diversity with increasing elevation, where surface air pressure decreases with altitude. However, it is still likely that other parameters affect microorganisms, as suggested by the change in bacterial diversity with elevation at Mount Fuji (Japan) (Singh et al., 2012). The highest bacterial diversity was observed at 2,500 m, along the tree line, and declined toward ∼3,700 m (near the summit), where extreme temperatures, UV radiation, and a lack of nutrients likely affected the microbial community more significantly than pressure changes. In addition, the Earth’s atmosphere is a unique ecosystem that enables the distribution of microorganisms (∼10^2^–10^5^ cells/mL in cloud or fog) through aerosolization (Delort et al., 2010; DasSarma and DasSarma, 2018). In the atmosphere, microorganisms have to contend with multiple hazards, including UV-C and cosmic radiation, low temperatures, desiccation, and oxidants (DasSarma and DasSarma, 2018), and it is unlikely that decreasing pressure plays the most significant role in microbial community diversity (Amato et al., 2007). Under these conditions, sporulation, resting stages, and biofilm formation are strategies used to withstand the multiple extremes (Delort et al., 2010).

### Radiation

Radiation sources include UV radiation, X-rays, gamma rays and more generally, cosmic rays. These different types of ionizing radiation, in particular UV and gamma rays, can impact microbial cells *via* direct and indirect (e.g., the formation of reactive oxygen species) mechanisms. The reactive oxygen species can then damage DNA, proteins, lipids, and RNA, in addition to initiating Fenton-type reactions within the cell due to the release of Fe^2+^ from Fe-S clusters (Webb and DiRuggiero, 2013). Radiation-resistant microorganisms have been shown to resist up to 30 kGy of γ-radiation, in the case of a thermophilic bacterium *Thermococcus gammatolerans* EJ3 (Jolivet et al., 2003) and a mesophilic bacterium *Deinococcus hohokamensis* (Rainey et al., 2005) and 100–1000 J/m^2^ of UV254, in a xerotolerant bacterium *Psychrobacter pacificensis* L0S3S-03b (La Duc et al., 2007). One of the first radiation-resistant microorganisms isolated was *Deinococcus radiodurans*, which has been well-studied and regarded as a model organism for understanding radiation tolerance (Krisko and Radman, 2013). Additionally, these microorganisms are often polyextremophiles (Table 4) (Fredrickson et al., 2008; Webb and DiRuggiero, 2013).

Many ecosystems on Earth are affected by some type of radiation, with the most extreme radiation emanating from human-made radioactive-contaminated sites. These range from 0.5 Bq/kg at the Great Lakes, United States (Trapeznikov, 1983) to 10^9^ Bq/kg at Hanford Site in Richland, WA, United States (Fredrickson et al., 2004). Radiation can additionally be found in subsurface environments, due to the radioactive decay of radiogenic isotopes (e.g., ^238^U, ^232^Th, and ^40^K), which could also be responsible for radiolytic hydrogen production (Dzaugis et al., 2016), potentially supporting *in situ* microbial productivity. Indeed, a hyperthermophilic and radiation-tolerant archaeon was isolated (*Thermococcus gammatolerans* EJ3) from a deep-sea hydrothermal environment located at the East Pacific Rise, where natural radioactivity occurs (^210^Pb, ^210^Po, ^222^Rn) (Jolivet et al., 2003).

There are several isolated microorganisms which can survive exposure to extreme radiation (kGy), including exposure to space conditions for hundreds of days (De Vera et al., 2012). UV radiation likely influenced the evolution of life, especially during the Archean, when the ozone layer had yet to develop in the upper atmosphere due to a lack of atmospheric O_2_. During this time, there were also intervals in which a photochemically produced organic haze would form, creating a UV shield (Arney et al., 2016). As such, the earliest life would have to contend with periods of intense UV radiation until enough O_2_ was produced by oxygenic phototrophs after the Great Oxidation Event (ca. 2.8–2.4 Ga). Through photochemical reactions at short UV radiation wavelengths (<242 nm), a protective ozone layer could be established, thus preventing a significant amount of short wavelength (<290 nm) radiation from penetrating to the surface (Caldwell et al., 1989; Phoenix et al., 2006). It is likely that microorganisms had to develop the necessary resistance to both UV and ionizing radiation. Indeed, model simulations demonstrate that the 200–300 nm wavelength range were several orders of magnitude higher about 4–3.5 Ga compared to current levels (Cnossen et al., 2007; Cockell and Raven, 2007), and as mentioned previously *T. gammatolerans* EJ3 was isolated from an environment with natural radioactivity (Jolivet et al., 2003). Microbial adaptions to radiation include more genome copies for genome redundancy (Anitori, 2012, chapter 2), changes in DNA repair functions (Byrne et al., 2014), a condensed nucleoid (Anitori, 2012, chapter 2), utilization of smaller amino acids (Sghaier et al., 2013), accumulation of Mn(II) (Daly et al., 2004), production of pigments (Mojib et al., 2013), and more, as described elsewhere (Confalonieri and Sommer, 2011; Anitori, 2012; Krisko and Radman, 2013). These adaptations are seen throughout the microbial tree of life; for example, two mutants of *Halobacterium* sp. NRC-1, which can tolerate extreme radiation levels (LD_50_ > 11 kGy), had upregulation of a protein complex known to be involved in DNA repair, replication and recombination (DeVeaux et al., 2007). This complex is closely related to the archaeal and eukaryotic protein known as RPA (replication protein A) and to the bacterial protein SSB (single-strand DNA-binding protein). Although radiation resistance has been observed throughout Archaea, Bacteria, and Eukarya, the origins and evolution of such adaptations to radiation has yet to be determined.

## Potential Expanded Ranges for Life

Earth’s ecosystems often have wider ranges for each of the environmental parameters considered in this review compared to the current known limits for life (Figure 2). As described in the previous sections, the physical and chemical conditions of Earth’s environments exhibit a wide range, much of which, but not all, has been shown to be exploited by microbial life. Since the first extremophile discoveries in 1969, each decade of exploration has broadened our view of the boundaries of microbial environmental habitability. Therefore, it is likely that the true limits of life have yet to be found. For example, observed limits for temperature are -20–130°C, the theoretical temperature limit is considered to be between -40 and 150°C due to decreasing metabolic rates at -40°C (∼100 million years to turn over all of the cellular carbon; Price and Sowers, 2004) and the denaturation of cellular components is at 150°C (Schulze-Makuch et al., 2017 and references therein). The ability of life to adapt and thrive under extreme conditions can be further supported by the analysis of the communities adapted to pH changes caused by human activity, including the dumping of mine drainage and steel slag. Earth’s natural ecosystems have a pH range of 0.02–12.5, but contaminated sites extend the range to pH -3.6–13.3 and have observable microbial communities (Méndez-García et al., 2015) (Table 2). Similar to pH, the current pressure range of microbial life (*P*_range_ 0.1–125 MPa) extends beyond that of Earth’s surface ecosystems (*P*_range_ 0.1–112 MPa), demonstrating life can resist more extreme values of both low and high pressure (see section “Pressure”). Similarly, microorganisms living in extreme salinity (salinity_life_ = 0–35%, salinity_Earth_ = 0–50%) also need to contend with water activity. As mentioned previously, the lowest a_w_ for life is currently estimated ∼0.611 a_w_ (Stevenson et al., 2015), but microbial life surpassed this water activity limit in DHABs (∼0.4 a_w_) (see section “Salinity and Water Activity”).

Although there are many (poly)extremophiles currently in culture (see Table 4 for some examples of notable polyextremophiles), data concerning the ability to withstand multiple stressors are extremely limited (Harrison et al., 2013). Moreover, the number of cultured microorganisms is tiny if compared to the diversity of uncultured clades (Hug et al., 2016). The number of uncultured microorganisms at the genus level has been recently estimated to be on average 7.3 × 10^29^, with ∼81% of microbial cells in environments such as the terrestrial subsurface, hypersaline environments, marine sediment, hot springs, and hydrothermal vents (Lloyd et al., 2018). These uncultivated microorganisms are very likely to include (poly)extremophiles and will aid in expanding our understanding of the boundary conditions of life.

## Can Life Originate, Evolve, or Survive on Other Planetary Bodies?

Different classification schemes have been published to describe planetary bodies based on their ‘habitability’ (e.g., Lammer et al., 2009; Noack et al., 2016; Schulze-Makuch et al., 2017). Several studies have also demonstrated the growth of microorganisms under lab-simulated planetary conditions, including Mars-like (Nicholson et al., 2013; Schuerger and Nicholson, 2016; Fajardo-Cavazos et al., 2018) and Enceladus-like (Taubner et al., 2018) conditions. In this context, defining the boundary limits of life on Earth is a crucial step in identifying the conditions likely to originate or support life on other planetary bodies. Therefore, studies on the limits of life are important to understand four areas: (1) the potential for panspermia, (2) forward contamination due to human exploration ventures, (3) planetary colonization by humans, and (4) the exploration of extinct and extant life. In this review, we outline the physical and chemical boundary conditions of Earth’s environments and those of life on Earth and compare them to the conditions observed on other planetary bodies in order to discuss whether life could originate, evolve, or survive elsewhere in our solar system and beyond.

Similar to Earth, other planetary bodies might have different environments with varying ranges for each parameter. Since our knowledge of individual niches or habitats is extremely limited for other planetary bodies, we considered the range of each parameter (temperature, salinity, pH, and pressure) across three planetary layers: (1) atmosphere, (2) surface, and (3) subsurface (Table 5). Many planetary bodies studied thus far have the potential for extinct or extant life, based on our knowledge of life on Earth. Depending on the planetary body, different (poly)extremophiles could persist. For example, halopsychrophiles might be able to persist on Titan, Ceres, and Europa, which likely have saline subsurface oceans (Grindrod et al., 2008; Zolotov and Kargel, 2009; Neveu and Desch, 2015), and also on Mars which could have Cl-rich subsurface brines (Clifford et al., 2010; Jones et al., 2011). These lifeforms would also need to withstand high pressures. For example, the hydrostatic pressure of the subsurface ocean at Titan ranges from 140 to 800 MPa (Sohl et al., 2014). While such pressures are beyond the range of the most extreme cultured piezophile on Earth (*Thermococcus piezophilus, P*_max_ = 125 MPa) (Dalmasso et al., 2016), microorganisms have successfully been exposed to pressures up to 2,000 MPa and found to be metabolically active in fluid inclusions within type-IV ice (Vanlint et al., 2011). Based on these observations it is possible that other planetary bodies may be within reach for Earth-based life (Table 5), including Enceladus (*P*_max_ = 50 MPa; Hsu et al., 2015) and Europa (*P*_max_ = 30 MPa; Muñoz-Iglesias et al., 2013).

**Table 5:** Boundary conditions for different planetary bodies of astrobiological interest (compared to Earth), split into atmosphere, surface, and subsurface layers.

Planetary	Type	Layer	Temperature	pH	Pressure	Salinity	Geochemistry	References
body			(°C)		(MPa)	(% NaCl)		
Earth	Planet	Atmosphere	-100 – 40	Neutral, local acidic conditions possible due to volcanism and human activities	0.0001–0.1	0	78% N_2_, 21% O_2_, 9340 ppm Ar, 400 ppm CO_2_ 18.2 ppm Ne, 5.2 ppm He, 1.7 ppm CH_4_, 1.1 ppm Kr, 0.6 ppm H_2_, variable H_2_O	Hans Wedepohl, 1995; McDonough and Sun, 1995; Wayne, 2000
		Surface	-98.6 – 464	-3.6 – 13.3	0.003–112	0–saturation	Soils and sediments of varying lithologies, siliceous crust, ranging from mafic to felsic composition. Extensive ocean (70% planet surface), with 4,000 m average depth, 4°C and 3.5% average temperature and salinity respectively	
		Subsurface	3.25–<400	∼1–12.8	<800	0.05–saturation	Soils and sediments of varying lithologies, siliceous crust, ranging from mafic to felsic composition, ultramafic mantle	
Venus	Planet	Atmosphere	-40 – 482^a^	0^b^	0.1–9.3^c^	nr	96.5% CO_2_, 3.5% N_2_; small quantities of CO, SO_2_, HCl, HF, HDO, and H_2_O; H_2_SO_4_ condensates	Cockell, 1999; Basilevsky and Head, 2003; Schulze-Makuch et al., 2004; Lang and Hansen, 2006; Bertaux et al., 2007; Airey et al., 2017
		Surface	377–482	nr	4.5–9.3^c^	nr	Rocks are similar to tholeiitic and alkaline basalts; no liquid water	
		Subsurface	nr	nr	nr	nr	Fluid channels; volcanism	
Mars	Planet	Atmosphere	-138 – 35^d^	nr	0.0001–0.0009	nr	95.3% CO_2_, 2.7% N_2_, 1.6% Ar, 0.13% O_2_, 0.08% CO; trace amounts of H_2_O, NO, Ne, Kr, Xe	Varnes et al., 2003; Fairén et al., 2004; Nicholson and Schuerger, 2005; Hecht et al., 2009; Smith et al., 2009; Johnson et al., 2011; Jones et al., 2011; Michalski et al., 2013; Longstaff, 2014; Wordsworth, 2016; Sinha et al., 2017; NASA, 2018
		Surface	-138 – 30	7.7^e^	0.0004–0.0009	5.2–5.8	Basaltic, Fe-/Mg-rich phyllosilicates, perchlorate salts, Al-rich clays, sulfates, chlorides, calcite, and silicas; potential cryosphere	
		Subsurface	55^g^	4.96–9.13^h^	10–303^g^	Cl-rich brines	Potential groundwater; basalt crust; possible serpentinization	
Enceladus	Icy moon	Plume jets	0	∼8.5–9	High velocity jets	>0.5	90–99% H_2_O, ≤0.61–4.27% N_2_, 0.3–5.3% CO_2_, 0.1–1.68% CH_4_, 0.4–0.9% NH_3_, 0.4–39% H_2_, trace amounts of hydrocarbons; high mass organic cations, silicates, sodium, potassium, carbonates	Gioia et al., 2007; Postberg et al., 2009, 2018; Waite et al., 2009; Zolotov et al., 2011; Glein et al., 2015; Holm et al., 2015; Hsu et al., 2015; Taubner et al., 2018
		Icy shell (∼10 km thick)	-233 – -23	nr	nr	May have ammonia brine pockets	May have tectonics	
		Subsurface global ocean (∼0–170 km depth)	<90	8.5–12.2^k^	1–8	0.45–<4	Possible serpentinization	
Titan	Icy moon	Atmosphere	-183 – -73^j^	nr	>0.01–0.15	nr	98.4% N_2_, 1.4% CH_4_, 0.2% H_2_, trace hydrocarbons and organics; ∼50 ppmv CO and ∼15 ppbv CO_2_; HCN, C_2_H_3_CN, and other nitriles; hazes and clouds	Fulchignoni et al., 2005; de Kok et al., 2007; Norman, 2011; Baland et al., 2014; Mastrogiuseppe et al., 2014; Mitri et al., 2014; Sohl et al., 2014; Jennings et al., 2016; McKay, 2016; Mitchell and Lora, 2016; Brassé et al., 2017; Cordier et al., 2017
		Surface	-183 – -179	nr	0.15–0.35^i^	nr	95% N_2_, 5% CH_4_, 0.1% H_2_; lakes and sea have CH_4_, C_2_H_4_, and dissolved nitrogen; dunes of solid organic material; low-latitude deserts and high-latitude moist climates	
		Subsurface	-18	11.8^l^	50–300^m^	Likely dense subsurface ocean (≤1,350 kg m^-3^) suggesting high salinity	CH_4_ and C_2_H_6_	
Ceres	Dwarf planet	Atmosphere	nr	nr	nr	nr	Transient atmosphere with possible water vapor	Fanale and Salvail, 1989; Zolotov, 2009, 2017; Küppers et al., 2014; Hayne and Aharonson, 2015; Neveu and Desch, 2015; Hendrix et al., 2016; Villarreal et al., 2017; Vu et al., 2017; Castillo-Rogez et al., 2018; McCord and Castillo-Rogez, 2018; McCord and Zambon, 2019
		Surface	(-157 – -30)^n^	9.7–11.3^n^	nr	<10^n^	Surface clays; (Mg, Ca)-carbonates; (Mg, NH_4_)-phyllosilicates; Fe-rich clays; salt deposits; chloride salts; water-rock interactions; brucite and magnetite; sulfur species and graphitized carbon; localized Na-carbonates (e.g., Na_2_CO_3_), NH_4_Cl, NH_4_HCO_3_	
		Subsurface	-143 – -93°	Likely alkaline	<140–200^p^	Potentially has briny or NH_3_-rich subsurface liquid	Active water/ice-driven subsurface processes	
Europa	Icy moon	Atmosphere (tenuous)	nr	nr	0.1^-12^–1^-12^	nr	Ion sputtering of the surface; potential water plumes; O_2_; trace amounts of sodium and potassium	Spencer et al., 1999; Chyba and Phillips, 2001; Marion et al., 2005; McGrath et al., 2009; Zolotov and Kargel, 2009;
								Travis et al., 2012; Cassidy et al., 2013; Muñoz-Iglesias et al., 2013; Kattenhorn and Prockter, 2014; Soderlund et al., 2014; Hand and Carlson, 2015; Kimura and Kitadai, 2015; Noell et al., 2015; Vance et al., 2016; Teolis et al., 2017; Zhu et al., 2017; Jones et al., 2018; Martin and McMinn, 2018; Pavlov et al., 2018
		Surface (icy shell)	-187 – -141	nr	0.1^-12^	May be saline, as delivered to the surface from a salty ocean, may have brine or salt inclusions	H_2_O_2_, H_2_SO_4_, CO_2_; salts concentrated in cracks; oxidants and simple organics; potentially MgSO_4_, Na_2_SO_4_, Na_2_CO_3_, may have gas inclusions; may have tectonics	
		Subsurface ocean	Daily inundation of seawater at *T* = -4 – 0	Potential for wide range^q^	0.1–30^r^	<3.5	Likely contains Mg^2+^, SO_4_^2-^, Na^+^, Cl^-^; oxidants and simple organics	

*The observed or putative geochemistry as well as other potential influences are also listed. ^*a*^Thermosphere can be as cold as -173°C (Bertaux et al., 2007); the upper-to-middle cloud layers are between -40 and 60°C (Cockell, 1999). ^*b*^Acid concentration in upper cloud layer is 81%, in lower layers up to 98% (Cockell, 1999). ^*c*^Up to 11 MPa in a deep depression (Basilevsky and Head, 2003). ^*d*^Summer air temperatures on Mars near the equator can reach a maximum of 35°C (Longstaff, 2014). ^*e*^Measured by the Phoenix Mars Lander Wet Chemistry Laboratory at the northern plains of the Vastitas Borealis (Hecht et al., 2009). ^*f*^Liquid water may have had water activity >0.95 (Fairén et al., 2009). ^*g*^Calculated temperature at a depth of 1–30 km (Jones et al., 2011; Sinha et al., 2017); at a depth ∼310 km, the calculated temperature is <427°C (Jones et al., 2011); the Martian core has temperature 1,527°C (Longstaff, 2014). ^*h*^Calculated groundwater pH (Varnes et al., 2003). ^*i*^Calculated pressure at Titan’s large sea, Ligeia Mare, is 0.20–0.35 MPa (Cordier et al., 2017). ^*j*^Tropospheric temperature can be -193°C; 80% of incident sunlight is absorbed by Titan’s atmosphere, suggesting that there are greenhouse and antigreenhouse effects (Mitchell and Lora, 2016). ^*k*^The subsurface ocean on Enceladus could also have pH range 10.8–13.5 (Glein et al., 2015). ^*l*^Calculated ocean pH with 5 wt% NH_3_ (Brassé et al., 2017). ^*m*^Calculated pressure for the subsurface ocean with thickness 100 km and outer shell thickness 40–170 km (Baland et al., 2014); 800 MPa at the mantle ice shell-core boundary (Sohl et al., 2014). ^*n*^Calculated surface temperatures, illuminated surfaces can have temperature <-173°C (Hayne and Aharonson, 2015); calculated pH and salinity for bright deposits in Occator crater (Zolotov, 2017); temperature for bright deposits in Occator crater might reach <-0.2°C (Zolotov, 2017). ^*o*^Internal temperature might reach 77°C (McCord and Sotin, 2005). ^*p*^Ceres’ center pressure (Zolotov, 2009). ^*q*^Acid brine may result from hydrothermal systems and be enriched with sulfuric acid (Kargel et al., 2000); neutral brine may occur as leachate from chondritic material and be enriched with magnesium sulfate (Kargel et al., 2000; Pasek and Greenberg, 2012); alkaline brine may occur in areas with natron (Na_2_CO_3_^⋅^10H_*2*_O), produced from the venting of CO_*2*_ from aqueous reservoirs (Langmuir, 1971; Millero and Rabindra, 1997). ^r^At the base of a 100 km Europan ocean, the pressure is calculated to be 146 MPa (Marion et al., 2005).*

The atmospheres of some planetary bodies could potentially harbor life as well. In particular, the upper-to-middle cloud layers of Venus (0–60°C; pH∼0) might be suitable for thermo- or psychro-acidophilic microorganisms (Table 4). Titan also has a dense atmosphere, but it is extremely cold (-183 – -78°C) and life on Earth can only metabolize at temperatures greater than -20°C (Rivkina et al., 2000). Other planetary bodies presented in Table 5 have transient or tenuous atmospheres that have extremely low pressures and likely cannot support life. In comparison, on Earth, microorganisms have been observed and cultured from the upper atmosphere, although stresses such as UV-C radiation, low temperatures, and oxidants make it difficult to survive (DasSarma and DasSarma, 2018). Microorganisms, in particular psychrophiles, with the capability of biofilm formation, clumping, and repair systems are more likely to tolerate Earth’s atmospheric conditions (DasSarma and DasSarma, 2018). Similar strategies may be needed on other planetary bodies.

The surface of other planetary bodies, such as Ceres, Europa, and Mars, experience high levels of radiation, and thus, may be unsuitable to support life. UV radiation is damaging for Earth-based life, and several studies have shown that there is a 99% loss in viability for microorganisms placed under Mars-like surface conditions, with UV-C as the most harmful source (Schuerger et al., 2003). However, shielding from UV-C radiation increases the chance of survival and includes shielding by atmospheric dust or burial (Barbier et al., 1998; Mancinelli and Klovstad, 2000; Cockell et al., 2002, 2005; Schuerger et al., 2003; Hansen et al., 2009; Johnson et al., 2011). Shielding is also necessary against charged particle radiation and can be achieved by burial at only centimeter depths below the surface. Indeed, the harsh radiation exposed to Europa’s surface inside the Jovian magnetosphere is predicted to only penetrate about 1–20 cm below the surface of Europa, as modeled by Nordheim et al. (2018).

Solar and galactic cosmic rays (high-energy particles with energies from 10 MeV to >10 GeV) present challenges to life on the surface and near-surface of Mars and other planetary bodies. However, any subsurface aquifer deeper than a few meters would be protected from damaging radiation. Dartnell et al. (2007) calculated the galactic cosmic ray dosage rates and the corresponding survival times (which they defined as a million-fold decrease in cell number) of characteristic microbes at different depths in Mars’s subsurface. At the surface, *E. coli* has a survival time of 1,200 years, while at 20-m depth, that survival time jumps to 1.5 × 10^8^ years. Compared to *E. coli, D. radiodurans* has survival times an order of magnitude longer. These survival times are, in fact, lower limits in light of recent measurements by the Radiation Assessment Detector onboard the Mars Science Laboratory (Hassler et al., 2014), which found that the actual dose rate at Gale Crater (76 mGy year^-1^) is a factor of 2 lower than that modeled by Dartnell et al. (2007).

With respect to cosmic rays, early Mars would have provided more favorable environments: Mars’s ancient dynamo may have produced a global magnetic field on the order of that of the present-day Earth (Weiss et al., 2008), and a thicker atmosphere would have also provided significantly impeded a flux of high-energy particles. Ehresmann et al. (2011) found that, with an atmosphere 100 times as thick, the dose rate at the surface decreases to ∼0.3–20% of its present-day value.

In addition to radiation, the surface of other planetary bodies is generally extremely cold. The minimum estimated temperature all planetary bodies listed in Table 5, except for Venus, ranges from -138°C (Mars) to -233°C (Enceladus) while the maximum estimated temperature ranges from -179°C (Titan) to 30°C (Mars). In accordance, the average temperature of satellites that are potential analogs to Earth is much less than -5°C, with many satellites likely experiencing wide temperature variation (Reid et al., 2006). This indicates the physiology of radiation-tolerant psychrophiles is important for understanding the potential of life on the surface of other planetary bodies, such as the production of a fibril network, cell aggregation, and cold shock proteins (Reid et al., 2006).

This suggests the subsurface is one of the most important locations in the search for extinct and extant extraterrestrial life (Jones et al., 2018). On Earth alone, the subsurface is estimated to house 50–87% of the Earth’s microorganisms (Kallmeyer et al., 2012; Magnabosco et al., 2018). The subsurface of other planetary bodies is potentially warmer than the surface and atmosphere (Table 5), influenced by geothermal processes [e.g., on Mars (Jones et al., 2011)], thermal convection [e.g., on Enceladus and Titan (Mitri and Showman, 2008)] and radiolysis [e.g., on Mars (Dzaugis et al., 2018)]. Several planetary bodies (Enceladus, Titan, Ceres, and Europa) likely have subsurface oceans, and Mars could potentially have a limited supply of groundwater (Clifford et al., 2010). Potential communities in these extraterrestrial subsurface environments are unlikely to be supported by surface exports of organic carbon like on our planet (Kallmeyer et al., 2012), but rather by *in situ* production fueled by H_2_ and abiotic CH_4_. The abiotic production of H_2_ can occur through a variety of mechanisms, including the radiolysis of water (Lin et al., 2005; Dzaugis et al., 2018) and serpentinization at both high and low temperatures (Neubeck et al., 2011; McCollom, 2016).

Serpentinization consists of water-rock interactions involving the hydration of Fe^2+^-rich minerals (primarily olivines), resulting in alkaline pH, production of H_2_ and potentially low-molecular weight organic carbon (e.g., formate, methane and a wide variety of other organic compounds) (Schrenk et al., 2013). Thus, serpentinization may have played a role in the origins of life on Earth (Russell et al., 2010) and perhaps on icy worlds as well (Russell et al., 2014, 2017). Several planetary bodies could have ongoing serpentinization in a subsurface ocean, including Enceladus, Titan, Ceres, and Europa (Table 5), and serpentinization reactions could be widespread in the cosmos (Holm et al., 2015). Mars might also have serpentinization occurring in the subsurface or had serpentinization occurring millions of years ago, as indicated by the observation of hydrated minerals, such as serpentine phases, on the surface of Mars (Ehlmann et al., 2010). Serpentinite-hosted sites on planetary bodies could likely support chemoautotrophic life, such as methanogens (McCollom, 1999). For example, the piezotolerant thermophile *Methanothermococcus okinawensis* was capable of growing under Enceladus-like conditions up to 5 MPa (Taubner et al., 2018), and the thermophilic methanogen, *Methanothermobacter wolfeii*, could survive subsurface Mars-like conditions across pH 5–9, pressure 0.1–122 MPa, and temperature at 55°C (Sinha et al., 2017). The 55°C temperature corresponds to a Martian depth of 1–30 km and 10–304 MPa (Sinha et al., 2017).

In contrast to serpentinization, radiolysis consists of radionuclides decay, such as uranium, thorium, and radioactive potassium, decomposing water molecules into oxidizing radicals that then react with oxidizable substrates, such as pyrite, generating the necessary chemical energy for life to survive. For example, the sulfate-reducing bacterium *Candidatus* Desulforudis audaxviator is the only species observed in fracture fluids at depths > 1.5 km (Mponeng mine, Johannesburg, South Africa) and is likely influenced by the radiolytic production of such chemical species as H_2_ and sulfate (Chivian et al., 2008). It is possible that radiolysis could support such life on other planetary bodies, including the Europan ocean (Altair et al., 2018) and the martian subsurface (Michalski et al., 2018).

It is important to note that the presence of liquid water (or other liquid solvent) is the main indicator to consider the possibility of extinct or extant life on a planetary body. Planetary bodies with low water activity (a_w_ < 0.6, see section “Salinity and Water Activity”) may not have the capability to harbor life. In places with low water activity, desiccation-tolerance could become an important factor in determining the survivability of organisms, coupled with the transient availability of water over time (either by precipitation, moisture, fog, or atmospheric humidity). For example, desiccation tolerant organisms may be able to survive under Mars-like surface conditions (Johnson et al., 2011).

While it is possible to use our knowledge of the boundary conditions of Earth’s life to map possible habitable environments on other planetary bodies, the discussion regarding the potential for life to originate elsewhere remains more elusive. Given the limited understanding of the processes that have led to life on our planet, discussions regarding the conditions under which life might originate on other planets remains rather speculative (McKay, 2014). As suggested previously (McKay, 2014), we might assume only planets possessing boundary conditions encompassing Earth’s biospace (Figure 2) and/or having all fundamental life requirements (e.g., energy source, solvent and building blocks) might be generative for life. An additional point to keep in mind while discussing the origin—and long-term persistence—of life on a planetary body is the necessity of elemental cycling on planetary scales (Jelen et al., 2016), a role often accomplished on our planet by a combination of geological and biological processes on our planet linked by a complex set of feedback processes over time (Chopra and Lineweaver, 2016; Moore et al., 2017).

## Future Directions and Outlook

Extremophiles have pushed our understanding of the boundaries of life in all directions since they were first discovered. As already highlighted by Harrison et al. (2013) and by the data presented in Tables 3, 4, an analysis of cultured extremophiles highlights that the majority of organisms in culture are in fact polyextremophiles. Despite this, there is a fundamental lack of studies addressing the tolerance of microorganisms to multiple extremes (Rothschild and Mancinelli, 2001; Harrison et al., 2013), potentially hindering our understanding of the limits of life. In the past 50 years of extremophile research it has become apparent that the limit of life varies when organisms face co-occurring multiple extremes. For example, the upper limit of life has been raised beyond 100°C when high pressure was also present (Stetter, 1982). Future research will need to focus more on the interaction factor between multiple parameters.

While considering the basic requirements of life discussed in the introduction (namely, energy, solvent, and building blocks), it is possible that the true limits of life are actually controlled by practical implications of these requirements. For example, the current theoretical limits of life regarding temperature, pressure, and salinity are directly linked to the water activity or the stability of biological molecules under such conditions (Price and Sowers, 2004). In the search of life’s true limits, it is therefore important to consider the effect (and combined effects) of any parameters directly controlling the availability of water, both at the community and subcellular level, and the stability of macromolecules.

The comparative and historical analysis of the limits of Earth’s life provides insight into the epistemology of life’s boundary research. Despite ongoing scientific investigations of our planet for most of recorded human history, we still find life in unexpected places, and given the number of Earth ecosystems that still need to be explored in detail, we expect the current boundary of life to be pushed even further. Comparing Earth’s parameter space with the biospace of Earth’s life (Figure 2), one can hypothesize that life might, indeed, have adapted to occupy nearly all available planetary niches, even transiently. Taken together, these observations suggest that the true shape of the terrestrial biosphere remains undefined. Moreover, the astonishing diversity of planetary bodies and exoplanets (Seager, 2013) will most likely expand the combinatorial space of environmental conditions, allowing us to speculate wildly about possible extraterrestrial lifeforms.

While considering the possibility for life to originate and exist on other planetary bodies, it is important to consider the variability of Earth local conditions when compared to the planetary mean (Tables 2, 5). The majority of parameters considered in this review are unlikely to be extreme over an entire planet, and local or transient conditions might still support life. An outstanding example are communities present in microbialites in the Atacama Desert, where seasonal water deliquescence on salt grains was sufficient to sustain a productive and diverse community (Davila et al., 2008). Similarly, Recurring Slope Lineae on the surface of Mars (McEwen et al., 2011) are an extraterrestrial example of a transient condition in which the presence of hydrated salts (Ojha et al., 2015) and seasonality suggests a role for water, albeit limited (Dundas et al., 2017). Therefore, it is unlikely that time-limited, coarse-grained observation of any extraterrestrial environment will be enough to definitely rule out the existence of life or conditions within the boundary space of Earth life, at least transiently.

Whether or not other planetary bodies such as Mars, Enceladus, or Europa could or did support life, the search for Earth’s life true limits will inform our exploration of space and could provide insight into processes that have led to the origin of life on our planet.

## Author Contributions

NM conducted literature search, created figures, and wrote the manuscript. HA, DB, JF-B, SZ, and MW conducted literature search and wrote the manuscript. DG devised the topic, supervised manuscript structure and data collection, conducted literature search, created figures, and wrote the manuscript.

## Conflict of Interest Statement

The authors declare that the research was conducted in the absence of any commercial or financial relationships that could be construed as a potential conflict of interest.
