# Corrigendum: Living at the Extremes: Extremophiles and the Limits of Life in a Planetary Context

**DOI:** 10.3389/fmicb.2019.01785

**Published:** 2019-08-13

**Authors:** Nancy Merino, Heidi S. Aronson, Diana P. Bojanova, Jayme Feyhl-Buska, Michael L. Wong, Shu Zhang, Donato Giovannelli

**Affiliations:** ^1^Department of Earth Sciences, University of Southern California, Los Angeles, CA, United States; ^2^Earth-Life Science Institute, Tokyo Institute of Technology, Tokyo, Japan; ^3^Biosciences and Biotechnology Division, Physical and Life Sciences Directorate, Lawrence Livermore National Lab, Livermore, CA, United States; ^4^Department of Biology, University of Southern California, Los Angeles, CA, United States; ^5^Department of Astronomy – Astrobiology Program, University of Washington, Seattle, WA, United States; ^6^NASA Astrobiology Institute's Virtual Planetary Laboratory, University of Washington, Seattle, WA, United States; ^7^Section of Infection and Immunity, Herman Ostrow School of Dentistry of USC, University of Southern California, Los Angeles, CA, United States; ^8^Department of Biology, University of Naples “Federico II”, Naples, Italy; ^9^Department of Marine and Coastal Science, Rutgers, The State University of New Jersey, New Brunswick, NJ, United States; ^10^Institute for Biological Resources and Marine Biotechnology, National Research Council of Italy, Ancona, Italy

**Keywords:** polyextremophiles, limits of life, astrobiology, habitability and astrobiology, extremophiles/extremophily, search for life

In the original article, there was a mistake in the legend for **Table 4** as published. The legend in **Table 4** is missing two parentheses around “Poly.” The correct legend appears below.

“**Table 4. Examples of notable (Poly)extremophiles and their physiological requirements**.”

Additionally, there was a mistake in Table 3 and Table 5 as published. In Table 3, the lowest temperature listed for *Planococcus halocryophilus* Or1 is “−18°C.” It should be “−15°C” instead. In addition, the pH range is “nr” but should be “6–11” instead. In the temperature column, 37 is bold type, but this should be regular type.

**Table 3:** Limits of life as identified by (poly)extremophilic organisms in pure cultures.

**Strain**	**Domain**	**Extremophile Type**	**Isolation ecosystem**	**Temperature (^**°**^C)**	**pH**	**Pressure (Mpa)**	**Salinity (%)**	**Water activity (a_**w**_)**	**References**
*Picrophilus oshimae* KAW 2/2	*Archaea*	Hypercidophile	Hot springs, Solfataras	47–65 (60)^a^	**−0.06–**1.8 (0.7)	nr	0–20	nr	Schleper et al., 1995, 1996
*Serpentinomonas* sp. B1	*Bacteria*	Alkaliphile	Serpentinizing system (water)	18–37 (30)	9–**12.5** (11)	nr	0–0.5 (0)	nr	Suzuki et al., 2014
*Methanopyrus kandleri* 116	*Archaea*	Hyperthemophile	Deep-sea hydrothermal vent	90–**122** (105)	(6.3–6.6)	0.4–40	0.5–4.5 (3.0)	nr	Takai et al., 2008
*Planococcus halocryophilus* Or1	*Bacteria*	Halopsychrophile	Sea ice core	**−15**–37 (25)	6–11 (7–8)	nr	0–19 (2)	nr	Mykytczuk et al., 2012, 2013
*Halarsenatibacter silvermanii* SLAS-1	*Bacteria*	Haloalkaliphile	Soda lake	28–55 (44)	8.7–9.8 (9.4)	nr	20–35 (**35**)	nr	Oremland et al., 2005
*Thermococcus piezophilus* CDGS	*Archaea*	Piezothermophile	Deep-sea hydrothermal vent	60–95 (75)	5.5–9 (6)	0.1–**125** (50)	2–6 (3)	nr	Dalmasso et al., 2016
Haloarchaeal strains GN-2 and GN-5	*Archaea*	Xerophile	Solar salterns (brine)	nr	nr	nr	nr	**0.635**	Javor, 1984

a *Data presented as range (optimum) for each parameter. nr, not reported in the original publication. Current limits are highlighted in bold*.

**Table 5:** Boundary conditions for different planetary bodies of astrobiological interest (compared to Earth), split into atmosphere, surface, and subsurface layers.

**Planetary body**	**Type**	**Layer**	**Temperature (^°^C)**	**pH**	**Pressure (MPa)**	**Salinity (% NaCl)**	**Geochemistry**	**References**
Earth	Planet	Atmosphere	−100 – 40	Neutral, local acidic conditions possible due to volcanism and human activities	0.0001 – 0.1	0	78% N_2_, 21% O_2_, 9340 ppm Ar, 400 ppm CO_2_ 18.2 ppm Ne, 5.2 ppm He, 1.7 ppm CH_4_, 1.1 ppm Kr, 0.6 ppm H_2_, variable H_2_	Hans Wedepohl, 1995; McDonough and Sun, 1995; Wayne, 2000
		Surface	−98.6 – 464	−3.6 – 13.3	0.003 – 112	0 – saturation	Soils and sediments of varying lithologies, siliceous crust, ranging from mafic to felsic composition. Extensive ocean (70% planet surface), with 4,000 m average depth, 4°C and 3.5% average temperature and salinity respectively	
		Subsurface	3.25 – <400	~1 – 12.8	<800	0.05 – saturation	Soils and sediments of varying lithologies, siliceous crust, ranging from mafic to felsic composition, ultramafic mantle	
Venus	Planet	Atmosphere	−40 – 482^a^	0^b^	0.1 – 9.3^c^	nr	96.5% CO_2_, 3.5% N_2_; small quantities of CO, SO_2_, HCl, HF, HDO, and H_2_O; H_2_SO_4_ condensates	Cockell, 1999; Basilevsky and Head, 2003; Schulze-Makuch et al., 2004; Lang and Hansen, 2006; Bertaux et al., 2007; Airey et al., 2017
		Surface	377 – 482	nr	4.5 – 9.3^c^	nr	Rocks are similar to tholeiitic and alkaline basalts; no liquid water	
		Subsurface	nr	nr	nr	nr	Fluid channels; volcanism	
Mars	Planet	Atmosphere	−138 – 35^d^	nr	0.0001–0.0009	nr	95.3% CO_2_, 2.7% N_2_, 1.6% Ar, 0.13% O_2_, 0.08% CO; trace amounts of H_2_O, NO, Ne, Kr, Xe	Varnes et al., 2003; Fairén et al., 2004; Nicholson and Schuerger, 2005; Hecht et al., 2009; Smith et al., 2009; Johnson et al., 2011; Jones et al., 2011; Michalski et al., 2013; Longstaff, 2014; Wordsworth, 2016; Sinha et al., 2017; NASA, 2018
		Surface	−138 – 30	7.7^e^	0.0004–0.0009	5.2–5.8	Basaltic, Fe-/Mg-rich phyllosilicates, perchlorate salts, Al-rich clays, sulfates, chlorides, calcite, and silicas; potential cryosphere	
		Subsurface	55^g^	4.96–9.13^h^	10–303^g^	Cl-rich brines	Potential groundwater; basalt crust; possible serpentinization	
Enceladus	Icy moon	Plume jets	0	~8.5 – 9	High velocity jets	> 0.5	90–99% H_2_O, ≤ 0.61–4.27% N_2_, 0.3–5.3% CO_2_, 0.1–1.68% CH_4_, 0.4–0.9% NH_3_, 0.4–39% H_2_, trace amounts of hydrocarbons; high mass organic cations, silicates, sodium, potassium, carbonates	Gioia et al., 2007; Postberg et al., 2009, 2018; Waite et al., 2009; Zolotov et al., 2011; Glein et al., 2015; Holm et al., 2015; Hsu et al., 2015; Taubner et al., 2018
		Icy shell (~10 km thick)	−233 – −23	nr	nr	May have ammonia brine pockets	May have tectonics	
		Subsurface global ocean (~0–170 km depth)	<90	8.5 – 12.2^k^	1 – 8	0.45 – <4	Possible serpentinization	
Titan	Icy moon	Atmosphere	−183 – −73^j^	nr	> 0.01 – 0.15	nr	98.4% N_2_, 1.4% CH_4_, 0.2% H_2_, trace hydrocarbons and organics; 95% N_2_, 5% CH_4_, 0.1% H_2_; ~50 ppmv CO and ~15 ppbv CO_2_; C_2_H_3_CN; clouds	Fulchignoni et al., 2005; de Kok et al., 2007; Norman, 2011; Baland et al., 2014; Mastrogiuseppe et al., 2014; Mitri et al., 2014; Sohl et al., 2014; Jennings et al., 2016; McKay, 2016; Mitchell and Lora, 2016; Brassé et al., 2017; Cordier et al., 2017
		Surface	−183 – −179	nr	0.15–0.35^i^	nr	Lakes and sea have CH_4_, C_2_H_4_, and dissolved nitrogen; dunes of solid organic material; low-latitude deserts and high-latitude moist climates	
		Subsurface	−18	11.8^l^	50–300^m^	Likely dense subsurface ocean (≤ 1,350 kg m^−3^) suggesting high salinity	CH_4_ and C_2_H_6_	
Ceres	Dwarf planet	Atmosphere	nr	nr	nr	nr	Transient atmosphere with possible water vapo	Fanale and Salvail, 1989; Zolotov, 2009, 2017; Küppers et al., 2014; Hayne and Aharonson, 2015; Neveu and Desch, 2015; Hendrix et al., 2016; Villarreal et al., 2017; Vu et al., 2017; Castillo-Rogez et al., 2018; McCord and Castillo-Rogez, 2018; McCord and Zambon, 2019
		Surface	(−157– −30)^n^	9.7–11.3^n^	nr	<10^n^	Surface clays; (Mg, Ca)-carbonates; (Mg, NH_4_)-phyllosilicates; Fe-rich clays; salt deposits; chloride salts; water-rock interactions; brucite and magnetite; sulfur species and graphitized carbon; localized Na-carbonates (e.g.,Na_2_CO_3_), NH_4_Cl, NH_4_HCO_3_	
		Subsurface	−143 – −93°^o^	Likely alkaline	<140 – 200^p^	Potentially has briny or NH_3_-rich subsurface liquid	Active water/ice-driven subsurface processes	
Europa	Icy moon	Atmosphere (tenuous)	nr	nr	0.1^−12^ – 1^−12^	nr	Ion sputtering of the surface; potential water plumes; O_2_; trace amounts of sodium and potassium	Spencer et al., 1999; Chyba and Phillips, 2001; Marion et al., 2005; McGrath et al., 2009; Zolotov and Kargel, 2009; Travis et al., 2012; Cassidy et al., 2013; Muñoz-Iglesias et al., 2013; Kattenhorn and Prockter, 2014; Soderlund et al., 2014; Hand and Carlson, 2015; Kimura and Kitadai, 2015; Noell et al., 2015; Vance et al., 2016; Teolis et al., 2017; Zhu et al., 2017; Jones et al., 2018; Martin and McMinn, 2018; Pavlov et al., 2018
		Surface (icy shell)	−187 - −141	nr	0.1^−12^	May be saline, as delivered to the surface from a salty ocean, may have brine or salt inclusions	H_2_O_2_, H_2_SO_4_, CO_2_; salts concentrated in cracks; oxidants and simple organics; potentially MgSO_4_, Na_2_SO_4_, Na_2_CO_3_, may have gas inclusions; may have tectonics	
		Subsurface ocean	Daily inundation of seawater at T = −4 – 0	Potential for wide range^q^	0.1 – 30^r^	<3.5	Likely contains Mg^2+^, ${\text{SO}}_{4}^{2-}$, Na^+^, Cl^−^; oxidants and simple organics	

The observed or putative geochemistry as well as other potential influences are also listed.

a Thermosphere can be as cold as −173°C (Bertaux et al., 2007); the upper-to-middle cloud layers are between −40 and 60°C (Cockell, 1999).

b Acid concentration in upper cloud layer is 81%, in lower layers up to 98% (Cockell, 1999).

c Up to 11 MPa in a deep depression (Basilevsky and Head, 2003).

d Summer air temperatures on Mars near the equator can reach a maximum of 35°C (Longstaff, 2014).

e Measured by the Phoenix Mars Lander Wet Chemistry Laboratory at the northern plains of the Vastitas Borealis (Hecht et al., 2009).

^f^ Liquid water may have had water activity > 0.95 (Fairén et al., 2009).

g Calculated temperature at a depth of 1–30 km (Jones et al., 2011; Sinha et al., 2017); at a depth ~310 km, the calculated temperature is <427°C (Jones et al., 2011); the Martian core has temperature 1527°C (Longstaff, 2014).

h Calculated groundwater pH (Varnes et al., 2003).

i Calculated pressure at Titan's large sea, Ligeia Mare, is 0.20–0.35 MPa (Cordier et al., 2017).

j Tropospheric temperature can be −193°C; 80% of incident sunlight is absorbed by Titan's atmosphere, suggesting that there are greenhouse and antigreenhouse effects (Mitchell and Lora, 2016).

k The subsurface ocean on Enceladus could also have pH range 10.8–13.5 (Glein et al., 2015).

l Calculated ocean pH with 5 wt% NH_3_ (Brassé et al., 2017).

m Calculated pressure for the subsurface ocean with thickness 100 km and outer shell thickness 40–170 km (Baland et al., 2014); 800 MPa at the mantle ice shell-core boundary (Sohl et al., 2014).

n Calculated surface temperatures, illuminated surfaces can have temperature < -173°C (Hayne and Aharonson, 2015); calculated pH and salinity for bright deposits in Occator crater (Zolotov, 2017); temperature for bright deposits in Occator crater might reach < -0.2°C (Zolotov, 2017).

o Internal temperature might reach 77°C (McCord and Sotin, 2005).

p Ceres' center pressure (Zolotov, 2009).

q Acid brine may result from hydrothermal systems and be enriched with sulfuric acid (Kargel et al., 2000); neutral brine may occur as leachate from chondritic material and be enriched with magnesium sulfate (Kargel et al., 2000; Pasek and Greenberg, 2012); alkaline brine may occur in areas with natron (Na_2_${\text{CO}}_{3}^{.}$10H_2_O), produced from the venting of CO_2_ from aqueous reservoirs (Langmuir, 1971; Millero and Rabindra, 1997).

r *At the base of a 100 km Europan ocean, the pressure is calculated to be 146 MPa (Marion et al., 2005)*.

In Table 5, the atmosphere entry for Earth > Atmosphere > Geochemistry is listed as “8.1% N_2_,” but the actual composition of Earth's atmosphere is “78% N_2._”

The corrected Table 3 and Table 5 appear below.

Lastly, there is a grammatical error in the original article.

A correction has therefore been made to the section Can ***Life Originate, Evolve, or Survive on Other Planetary Bodies?***, paragraph five:

“Solar and galactic cosmic rays (high-energy particles with energies from 10 MeV to >10 GeV) present challenges to life on the surface and near-surface of Mars and other planetary bodies. However, any subsurface aquifer deeper than a few meters would be protected from damaging radiation. Dartnell et al. (2007) calculated the galactic cosmic ray dosage rates and the corresponding survival times (which they defined as a million-fold decrease in cell number) of characteristic microbes at different depths in Mars's subsurface. At the surface, *E. coli* has a survival time of 1,200 years, while at 20-m depth, that survival time jumps to 1.5 × 10^8^ years. Compared to *E. coli, D. radiodurans* has survival times an order of magnitude longer. These survival times are, in fact, lower limits in light of recent measurements by the Radiation Assessment Detector onboard the Mars Science Laboratory (Hassler et al., 2014), which found that the actual dose rate at Gale Crater (76 mGy year^−1^) is a factor of 2 lower than that modeled by Dartnell et al. (2007).”

The authors apologize for these errors and state that they do not change the scientific conclusions of the article in any way. The original article has been updated.
